# The genome sequence of the Rock-rose Pot Beetle, *Cryptocephalus primarius *Harold, 1872

**DOI:** 10.12688/wellcomeopenres.23703.1

**Published:** 2025-02-19

**Authors:** Ryan Mitchell, Michael F. Geiser, Toby Turner, Natural History Museum Genome Acquisition Lab, Darwin Tree of Life Barcoding collective, Wellcome Sanger Institute Tree of Life Management, Samples and Laboratory team, Wellcome Sanger Institute Scientific Operations: Sequencing Operations, Wellcome Sanger Institute Tree of Life Core Informatics team, Tree of Life Core Informatics collective, Darwin Tree of Life Consortium

**Affiliations:** 1 Independent researcher, Sligo, County Sligo, Ireland; 2 Natural History Museum, London, England, UK

**Keywords:** Cryptocephalus primarius, Rock-rose Pot Beetle, genome sequence, chromosomal, Coleoptera

## Abstract

We present a genome assembly from a male specimen of *Cryptocephalus primarius* (Rock-rose Pot Beetle; Arthropoda; Insecta; Coleoptera; Chrysomelidae). The genome sequence has a total length of 370.99 megabases. Most of the assembly (87.88%) is scaffolded into 21 chromosomal pseudomolecules, including the X and Y sex chromosomes. The mitochondrial genome has also been assembled and is 17.97 kilobases in length. Gene annotation of this assembly on Ensembl identified 10,661 protein-coding genes.

## Species taxonomy

Eukaryota; Opisthokonta; Metazoa; Eumetazoa; Bilateria; Protostomia; Ecdysozoa; Panarthropoda; Arthropoda; Mandibulata; Pancrustacea; Hexapoda; Insecta; Dicondylia; Pterygota; Neoptera; Endopterygota; Coleoptera; Polyphaga; Cucujiformia; Chrysomeloidea; Chrysomelidae; Cryptocephalinae; *Cryptocephalu*s; *Cryptocephalus primarius* Harold, 1872 (NCBI:txid1587206)

## Background

*Cryptocephalus primarius* Harold, 1872, commonly known as the Rock-rose Pot Beetle, is a member of the leaf beetle family, Chrysomelidae, one of the largest clades of Coleoptera counting over 40,000 described species worldwide, of which about 250 occur in the British Isles ( Duff, 2018). Cryptocephalinae, the “case bearing leaf beetles” are one of the major subfamilies, sharing a distinctive body shape and an interesting biology: Their larvae are known to construct a hard protective case out of their own faecal matter, into which they can retreat ( Jolivet, 1997). *Cryptocephalus* Geoffroy, 1762 is by far the most species-rich genus of the entire Chrysomelidae, comprising over 1800 valid species worldwide, found in all biogeographic regions ( Bezděk & Sekerka, 2024; Schöller, 2002). In Britain, 22 species are present, of which two were only added in 2019 ( Duff, 2018; Telfer, 2019a; Telfer, 2019b). The subgeneric classification of *Cryptocephalus* is still very unsatisfactory, with numerous subgenera erected and later synonymised ( Schöller, 2021). At present, seven subgenera are still recognised as valid ( Bezděk & Sekerka, 2024), of which three are represented in Britain ( Duff, 2018). *C. primarius* is currently classified within *Cryptocephalus* s. str. This is the second data note to be published for a species of this group; the full genome of *Cryptocephalus moraei* was made available recently ( Sivell *et al.*, 2023).

*Cryptocephalus* are easily recognisable among other British Chrysomelidae by their characteristic “box like” body shapes with their small heads sunken into the prothorax and usually not visible from above. As members of Cryptocephalini, they have relatively long, filiform antennae, rather than short and serrate antennae like members of Clytrini, e.g. *Clytra quadripunctata* (Linnaeus, 1758). *C. primarius* is the largest of the British *Cryptocephalus* species, measuring 5.3–7.6 mm ( Duff, 2016). The ladybird-like elytral pattern is very distinctive, consisting of five round spots on red background on each elytron. Three of these spots are located close to the outer margin, one on the posterior part of the disc, one in the anterior part not far from the scutellum. The pronotum, legs and outer half of the antennae are completely black. Unlike other red and black coloured *Cryptocephalus* like *C. bipunctatus* (Linnaeus, 1758), the elytral punctures in *C. primarius* are completely irregular, not arranged into rows ( Duff, 2016). Varieties of *C. coryli* (Linnaeus, 1758) have a pattern somewhat resembling that of *C. primarius*, but that species has at least the outer margins of the black pronotum narrowly lined with red (males), and a completely red pronotum in the females ( Warchałowski, 2010).

*C. primarius* is a western European species, recorded from the UK, Portugal, Spain, France, Italy, Switzerland, Belgium, Germany and Czech Republic ( Bezděk & Sekerka, 2024). Throughout its range, it is considered a rare or very rare species. In Belgium, only two localities are known ( Fagot, 2020). In Germany and Czech Republic, it is listed as endangered ( Rheinheimer & Hassler, 2018; Sekerka *et al.*, 2017). Its occurrences in Britain represent the northernmost edge of its range. Within Britain, *C. primarius* is known from a small handful of sites in England, plus a 19th century record from Rannoch in Scotland, where it is now presumed to be extinct ( Cox, 2007; Wiltshire & Owen, 2004). It is listed as a “nationally rare” and critically endangered species, not only due to its tiny area of occupancy (estimated as less than 4 km ^2^), but also because of its declining population trend, with several historical populations now extinct ( Cox, 2007; Hubble, 2014). The major threats to the conservation of this species are habitat loss and degradation by succession of vegetation via neglect and arable conversion methods (i.e. seeding/fertiliser application) ( Hubble, 2014).

*C. primarius* was previously localised in six sites in Gloucestershire, Berkshire, Cambridgeshire and Dorset but, in 2004, seemed to have survived only in a single locality, Stinchcombe Hill in Gloucestershire ( Wiltshire & Owen, 2004). Ten years later, two undisclosed sites on the Dorset coast were mentioned ( Hubble, 2014). After Natural England’s review of this species ( Hubble, 2014) organisations such as Buglife, Butterfly conservation UK and BftB (Back from the Brink) have collaborated to ensure its survival in England. Species monitoring was carried out by Buglife and its volunteers through annual surveying of sites with recent records of *C. primarius* between 2018 to 2021. With thanks the efforts of Buglife and their volunteers, the species survived at Stinchcombe Hill and was re-discovered at another Gloucestershire site, Rodborough Common, after 35 years ( Back from the Brink, 2022). Surprisingly, the most recent NBN map shows several additional recent records, including some from Dorset, Wiltshire, the Isle of Wight and Norfolk ( NBN Atlas Partnership, 2024). Some need to be treated with caution, such as the unconfirmed sightings and the record apparently in the middle of the English Channel, others may suggest that the species has had a slight resurgence in recent years, or more thorough recording has led to discoveries of previously overlooked populations.

In Britain *C. primarius* is associated with unimproved chalk grassland on warm, south-facing slopes as a preferred microclimate. It feeds exclusively on Rock-roses ( *Helianthemum* spp.), in Britain only on *H. nummularium*, in other parts of its range also on a handful of related species. Adults feed on petals, anthers and pollen while larvae prefer stems and leaves ( Cox, 2007; Rheinheimer & Hassler, 2018). It is emphasised that carefully managed grazing is paramount for the continuity of *C. primarius* habitats in Britain, as habitat succession threatens populations of its host plant, *H. nummularium*. Alternatively in cases where grazing is untenable, manual removal of scrub is required to counteract encroachment ( Back from the Brink, 2022).

*C. primarius* is a univoltine species with rather short-lived adults, occurring between mid-May and late June, with peak abundance between 29 May and 5 June ( Wiltshire & Owen, 2004). Its larval biology was studied by Owen (2005), although the larval morphology has still not been formally described ( Cox, 2007).

**Figure 1.**
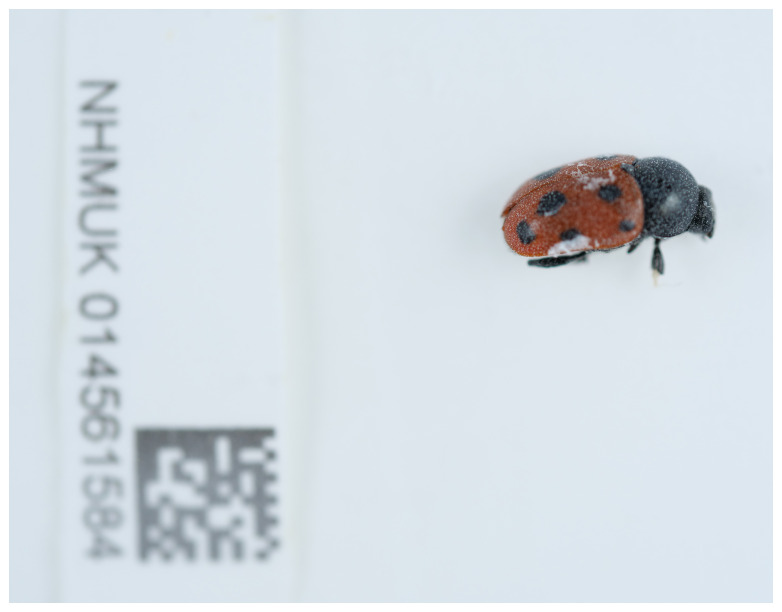
Photograph of the *Cryptocephalus primarius* (icCryPrim1) specimen used for genome sequencing.

The genome of *Cryptocephalus primarius* was sequenced as part of the Darwin Tree of Life Project, a collaborative effort to sequence all named eukaryotic species in the Atlantic Archipelago of Britain and Ireland. Here we present a chromosome-level genome sequence for *Cryptocephalus primarius*, based on a male specimen from Swanage Bay, England., England, United Kingdom ( Figure 1).

## Genome sequence report

### Sequencing data

The genome of a specimen of *Cryptocephalus primarius* ( Figure 1) was sequenced using Pacific Biosciences single-molecule HiFi long reads, generating 21.63 Gb from 2.52 million reads. GenomeScope analysis of the PacBio HiFi data estimated the haploid genome size at 318.02 Mb, with a heterozygosity of 0.26% and repeat content of 27.05%. These values provide an initial assessment of genome complexity and the challenges anticipated during assembly. Based on this estimated genome size, the sequencing data provided approximately 65.0x coverage of the genome. Chromosome conformation Hi-C sequencing produced 99.80 Gb from 660.92 million reads. Table 1 summarises the specimen and sequencing information, including the BioProject, study name, BioSample numbers, and sequencing data for each technology.

**Table 1.** Specimen and sequencing data for *Cryptocephalus primarius*.

Project information			
**Study title**	Cryptocephalus primarius			
**Umbrella BioProject**	PRJEB56483			
**Species**	*Cryptocephalus primarius*			
**BioSpecimen**	SAMEA14448122			
**NCBI taxonomy ID**	1587206			
Specimen information			
**Technology**	**ToLID**	**BioSample accession**	**Organism part**
**PacBio long read sequencing**	icCryPrim1	SAMEA14448158	whole organism
**Hi-C sequencing**	icCryPrim1	SAMEA14448158	whole organism
Sequencing information			
**Platform**	**Run accession**	**Read count**	**Base count (Gb)**
**Hi-C Illumina NovaSeq 6000**	ERR10323132	6.61e+08	99.8
**PacBio Sequel IIe**	ERR10357393	2.52e+06	21.63

### Assembly statistics

The primary haplotype was assembled, and contigs corresponding to an alternate haplotype were also deposited in INSDC databases. The assembly was improved by manual curation, which corrected 80 misjoins or missing joins and removed 6 haplotypic duplications. These interventions reduced the total assembly length by 0.72%, decreased the scaffold count by 17.12%, and increased the scaffold N50 by 12.75%. The final assembly has a total length of 370.99 Mb in 304 scaffolds, with 245 gaps, and a scaffold N50 of 15.6 Mb ( Table 2).

**Table 2.** Genome assembly data for *Cryptocephalus primarius*.

Genome assembly		
Assembly name	icCryPrim1.1	
Assembly accession	GCA_963576515.1	
*Alternate haplotype accession*	*GCA_963576505.1*	
Assembly level for primary assembly	chromosome	
Span (Mb)	370.99	
Number of contigs	549	
Number of scaffolds	304	
Longest scaffold (Mb)	33.5	
Assembly metric	Measure	*Benchmark*
Contig N50 length	1.87 Mb	*≥ 1 Mb*
Scaffold N50 length	15.6 Mb	*= chromosome N50*
Consensus quality (QV)	Primary: 59.0; alternate: 63.7; combined 59.6	*≥ 40*
*k*-mer completeness	Primary: 99.17%; alternate: 19.03%; combined: 99.48%	*≥ 95%*
BUSCO *	C:99.1%[S:98.0%,D:1.1%], F:0.4%,M:0.5%,n:2,124	*S > 90%; D < 5%*
Percentage of assembly mapped to chromosomes	87.57%	*≥ 90%*
Sex chromosomes	X and Y	*localised homologous pairs*
Organelles	Mitochondrial genome: 17.97 kb	*complete single alleles*
**Genome annotation of assembly GCA_963576515.1 at Ensembl**		
Number of protein-coding genes	10,661	
Number of non-coding genes	869	
Number of gene transcripts	17,076	

* BUSCO scores based on the endopterygota_odb10 BUSCO set using version 5.5.0. C = complete [S = single copy, D = duplicated], F = fragmented, M = missing, n = number of orthologues in comparison.

**Figure 2.**
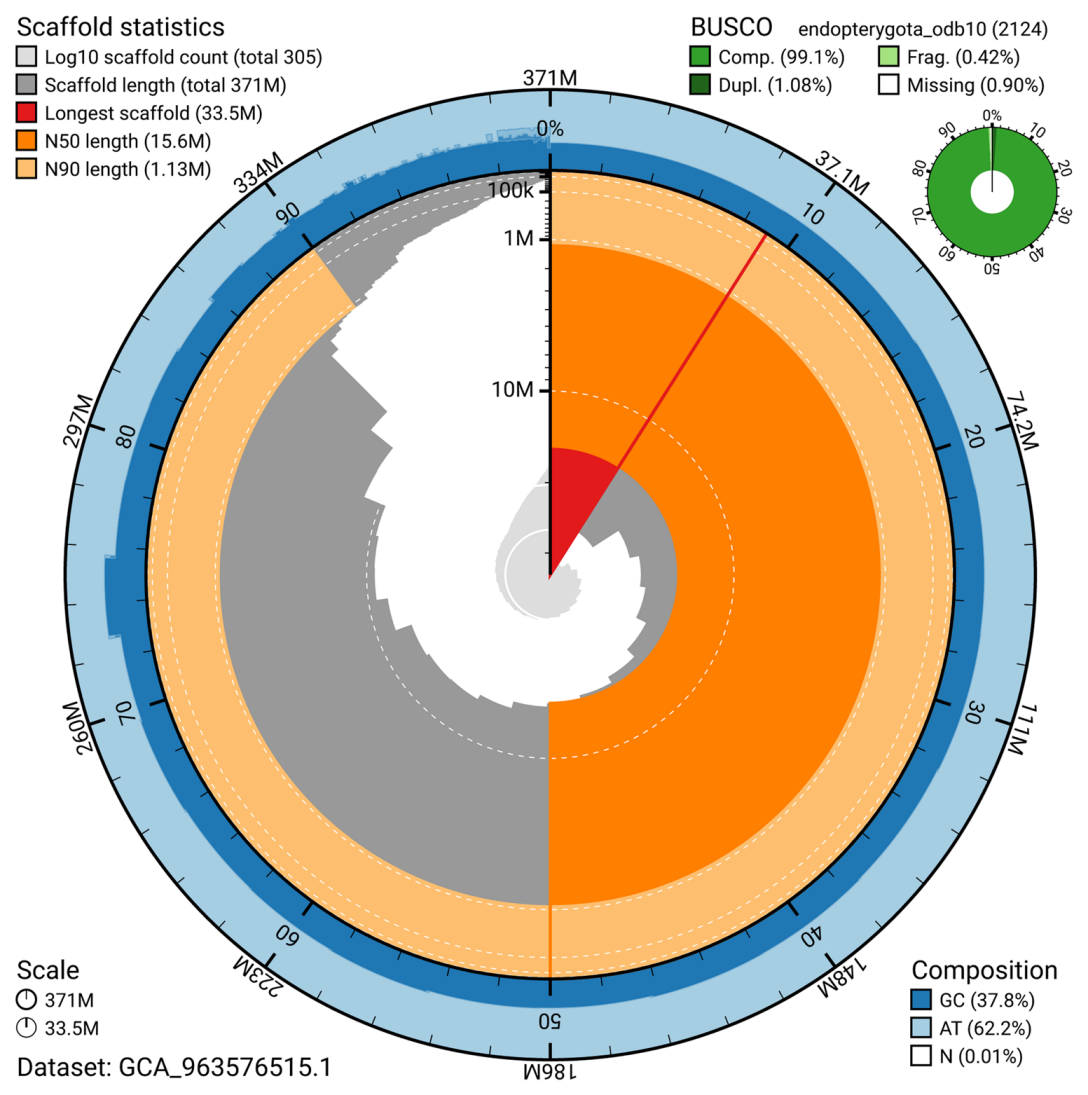
Genome assembly of *Cryptocephalus primarius*, icCryPrim1.1: metrics. The BlobToolKit snail plot provides an overview of assembly metrics and BUSCO gene completeness. The circumference represents the length of the whole genome sequence, and the main plot is divided into 1,000 bins around the circumference. The outermost blue tracks display the distribution of GC, AT, and N percentages across the bins. Scaffolds are arranged clockwise from longest to shortest and are depicted in dark grey. The longest scaffold is indicated by the red arc, and the deeper orange and pale orange arcs represent the N50 and N90 lengths. A light grey spiral at the centre shows the cumulative scaffold count on a logarithmic scale. A summary of complete, fragmented, duplicated, and missing BUSCO genes in the endopterygota_odb10 set is presented at the top right. An interactive version of this figure is available at https://blobtoolkit.genomehubs.org/view/GCA_963576515.1/dataset/GCA_963576515.1/snail.

**Figure 3.**
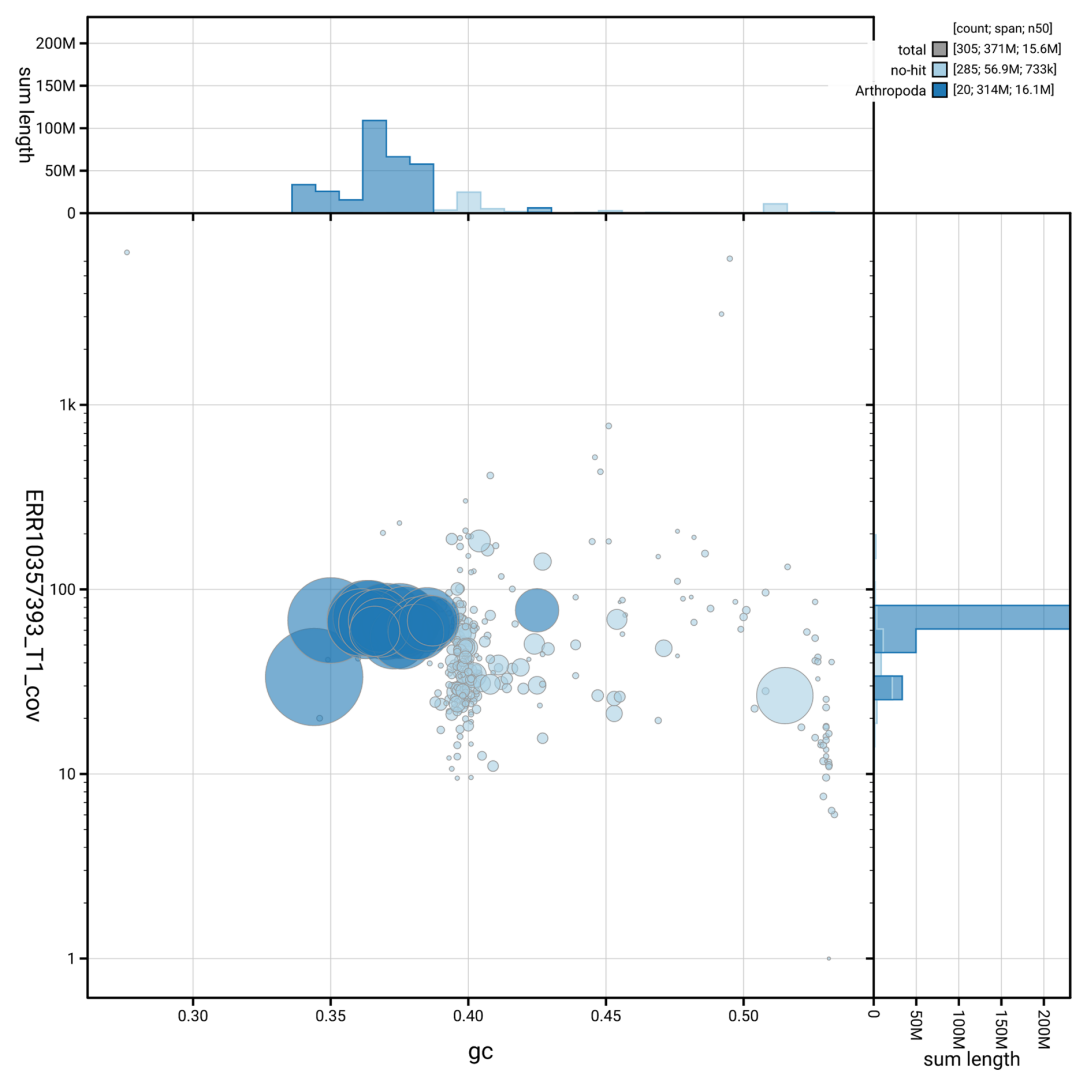
Genome assembly of *Cryptocephalus primarius*, icCryPrim1.1: BlobToolKit GC-coverage plot. Blob plot showing sequence coverage (vertical axis) and GC content (horizontal axis). The circles represent scaffolds, with the size proportional to scaffold length and the colour representing phylum membership. The histograms along the axes display the total length of sequences distributed across different levels of coverage and GC content. An interactive version of this figure is available at https://blobtoolkit.genomehubs.org/view/GCA_963576515.1/blob.

**Figure 4.**
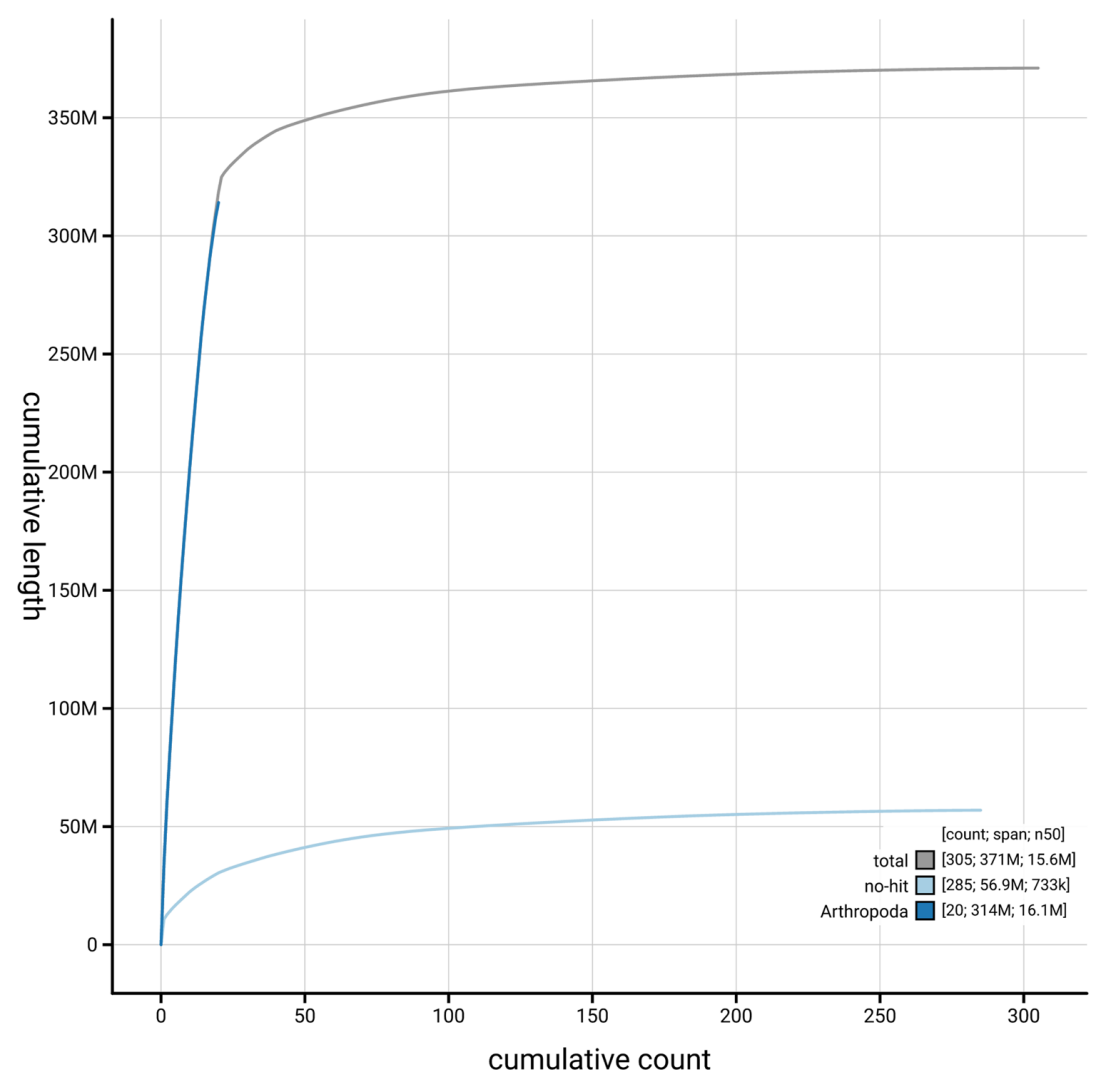
Genome assembly of *Cryptocephalus primarius,* icCryPrim1.1: BlobToolKit cumulative sequence plot. The grey line shows cumulative length for all scaffolds. Coloured lines show cumulative lengths of scaffolds assigned to each phylum using the buscogenes taxrule. An interactive version of this figure is available at https://blobtoolkit.genomehubs.org/view/GCA_963576515.1/dataset/GCA_963576515.1/cumulative.

The snail plot in Figure 2 provides a summary of the assembly statistics, indicating the distribution of scaffold lengths and other assembly metrics. Figure 3 shows the distribution of scaffolds by GC proportion and coverage. Figure 4 presents a cumulative assembly plot, with separate curves representing different scaffold subsets assigned to various phyla, illustrating the completeness of the assembly.

**Figure 5.**
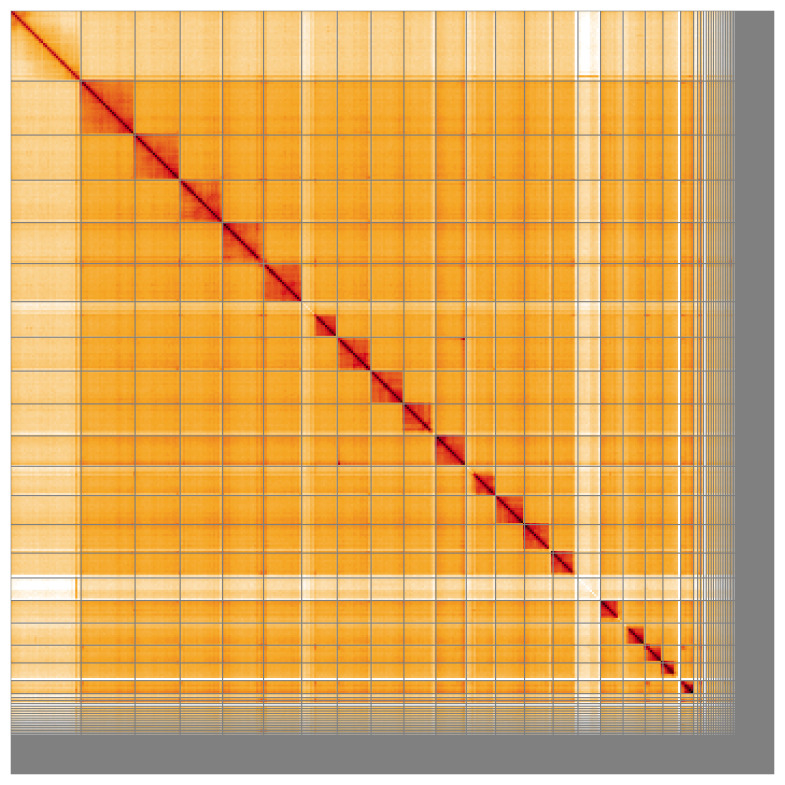
Genome assembly of *Cryptocephalus primarius:* Hi-C contact map of the icCryPrim1.1 assembly, visualised using HiGlass. Chromosomes are shown in order of size from left to right and top to bottom. An interactive version of this figure may be viewed at https://genome-note-higlass.tol.sanger.ac.uk/l/?d=fNUP0QxqTgGKu5FOYYhujg.

Most of the assembly sequence (87.57%) was assigned to 21 chromosomal-level scaffolds, representing 19 autosomes and the X and Y sex chromosome. These chromosome-level scaffolds, confirmed by Hi-C data, are named according to size ( Figure 5; Table 3). During curation, chromosomes X and Y were assigned by read coverage statistics. There is a large proportion of unplaceable subtelomeric repeat sequence.

**Table 3.** Chromosomal pseudomolecules in the genome assembly of *Cryptocephalus primarius*, icCryPrim1.

INSDC accession	Name	Length (Mb)	GC%
OY754937.1	1	25.67	35
OY754938.1	2	21.43	36.5
OY754939.1	3	20.19	37
OY754940.1	4	19.5	37.5
OY754941.1	5	18.11	36.5
OY754942.1	6	16.9	37.5
OY754943.1	7	16.12	37.5
OY754944.1	8	15.6	36
OY754945.1	9	15.23	36.5
OY754946.1	10	14.46	38.5
OY754947.1	11	13.91	37.5
OY754948.1	12	13.84	37
OY754949.1	13	13.55	38
OY754950.1	14	11.84	37
OY754952.1	15	10.72	38.5
OY754953.1	16	10.47	38
OY754954.1	17	8.45	38.5
OY754955.1	18	8.41	36.5
OY754956.1	19	6.22	42.5
OY754936.1	X	33.5	34.5
OY754951.1	Y	10.76	51.5
OY754957.1	MT	0.02	27.5

The mitochondrial genome was also assembled. This sequence is included as a contig in the multifasta file of the genome submission and as a standalone record in GenBank.

### Assembly quality metrics

The estimated Quality Value (QV) and *k*-mer completeness metrics, along with BUSCO completeness scores, were calculated for each haplotype and the combined assembly. The QV reflects the base-level accuracy of the assembly, while *k*-mer completeness indicates the proportion of expected *k*-mers identified in the assembly. BUSCO scores provide a measure of completeness based on benchmarking universal single-copy orthologues.

The primary haplotype has a QV of 59.0, and the combined primary and alternate assemblies achieve an estimated QV of 59.6. The *k*-mer completeness for the primary haplotype is 99.17%, and for the alternate haplotype it is 19.03%. The combined primary and alternate assemblies achieve a *k*-mer completeness of 99.48%. BUSCO analysis using the endopterygota_odb10 reference set ( *n* = 2,124) indicated a completeness score of 99.1% (single = 98.0%, duplicated = 1.1%).

Table 2 provides assembly metric benchmarks adapted from Rhie *et al.* (2021) and the Earth BioGenome Project Report on Assembly Standards September 2024. The achieves the EBP reference standard of 6.7.Q59.

## Genome annotation report

The *Cryptocephalus primarius* genome assembly (GCA_963576515.1) was annotated at the European Bioinformatics Institute (EBI) on Ensembl Rapid Release. The resulting annotation includes 17,076 transcribed mRNAs from 10,661 protein-coding and 869 non-coding genes ( Table 2; https://rapid.ensembl.org/Cryptocephalus_primarius_GCA_963576515.1/Info/Index). The average transcript length is 8,265.52. There are 1.48 coding transcripts per gene and 5.52 exons per transcript.

## Methods

### Sample acquisition and DNA barcoding

An adult male *Cryptocephalus primarius* (specimen ID NHMUK014561584, ToLID icCryPrim1) was collected from Swanage Bay, England., England, United Kingdom (latitude 50.61, longitude –1.95) on 2021-05-15. The specimen was collected and identified by Ryan Mitchell (Oumnh) and preserved by dry freezing (–80 °C).

The initial identification by morphology was verified by an additional DNA barcoding process according to the framework developed by Twyford *et al*. (2024). A small sample was dissected from the specimen and stored in ethanol, while the remaining parts were shipped on dry ice to the Wellcome Sanger Institute (WSI) ( Pereira *et al.*, 2022). The tissue was lysed, the COI marker region was amplified by PCR, and amplicons were sequenced and compared to the BOLD database, confirming the species identification ( Crowley *et al.*, 2023). Following whole genome sequence generation, the relevant DNA barcode region was also used alongside the initial barcoding data for sample tracking at the WSI ( Twyford *et al.*, 2024). The standard operating procedures for Darwin Tree of Life barcoding have been deposited on protocols.io ( Beasley *et al.*, 2023).

Metadata collection for samples adhered to the Darwin Tree of Life project standards described by Lawniczak *et al*. (2022).

### Nucleic acid extraction

The workflow for high molecular weight (HMW) DNA extraction at the Wellcome Sanger Institute (WSI) Tree of Life Core Laboratory includes a sequence of procedures: sample preparation and homogenisation, DNA extraction, fragmentation and purification. Detailed protocols are available on protocols.io ( Denton *et al.*, 2023b). The icCryPrim1 sample was prepared for DNA extraction by weighing and dissecting it on dry ice ( Jay *et al.*, 2023). Tissue from the whole organism was homogenised using a PowerMasher II tissue disruptor ( Denton *et al.*, 2023a). HMW DNA was extracted in the WSI Scientific Operations core using the Automated MagAttract v2 protocol ( Oatley *et al.*, 2023). The DNA was sheared into an average fragment size of 12–20 kb in a Megaruptor 3 system ( Bates *et al.*, 2023). Sheared DNA was purified by solid-phase reversible immobilisation, using AMPure PB beads to eliminate shorter fragments and concentrate the DNA ( Strickland *et al.*, 2023). The concentration of the sheared and purified DNA was assessed using a Nanodrop spectrophotometer and Qubit Fluorometer using the Qubit dsDNA High Sensitivity Assay kit. Fragment size distribution was evaluated by running the sample on the FemtoPulse system.

### Hi-C sample preparation

Tissue from the whole organism of the icCryPrim1 sample was processed for Hi-C sequencing at the WSI Scientific Operations core, using the Arima-HiC v2 kit. In brief, 20–50 mg of frozen tissue (stored at –80 °C) was fixed, and the DNA crosslinked using a TC buffer with 22% formaldehyde concentration. After crosslinking, the tissue was homogenised using the Diagnocine Power Masher-II and BioMasher-II tubes and pestles. Following the Arima-HiC v2 kit manufacturer's instructions, crosslinked DNA was digested using a restriction enzyme master mix. The 5’-overhangs were filled in and labelled with biotinylated nucleotides and proximally ligated. An overnight incubation was carried out for enzymes to digest remaining proteins and for crosslinks to reverse. A clean up was performed with SPRIselect beads prior to library preparation. Additionally, the biotinylation percentage was estimated using the Qubit Fluorometer v4.0 (Thermo Fisher Scientific) and Qubit HS Assay Kit and Arima-HiC v2 QC beads.

### Library preparation and sequencing

Library preparation and sequencing were performed at the WSI Scientific Operations core.

***PacBio HiFi***

At a minimum, samples were required to have an average fragment size exceeding 8 kb and a total mass over 400 ng to proceed to the low input SMRTbell Prep Kit 3.0 protocol (Pacific Biosciences, California, USA), depending on genome size and sequencing depth required. Libraries were prepared using the SMRTbell Prep Kit 3.0 (Pacific Biosciences, California, USA) as per the manufacturer's instructions. The kit includes the reagents required for end repair/A-tailing, adapter ligation, post-ligation SMRTbell bead cleanup, and nuclease treatment. Following the manufacturer’s instructions, size selection and clean up was carried out using diluted AMPure PB beads (Pacific Biosciences, California, USA). DNA concentration was quantified using the Qubit Fluorometer v4.0 (Thermo Fisher Scientific) with Qubit 1X dsDNA HS assay kit and the final library fragment size analysis was carried out using the Agilent Femto Pulse Automated Pulsed Field CE Instrument (Agilent Technologies) and gDNA 55kb BAC analysis kit.

Samples were sequenced using the Sequel IIe system (Pacific Biosciences, California, USA). The concentration of the library loaded onto the Sequel IIe was in the range 40–135 pM. The SMRT link software, a PacBio web-based end-to-end workflow manager, was used to set-up and monitor the run, as well as perform primary and secondary analysis of the data upon completion.

***Hi-C***

For Hi-C library preparation, DNA was fragmented using the Covaris E220 sonicator (Covaris) and size selected using SPRISelect beads to 400 to 600 bp. The DNA was then enriched using the Arima-HiC v2 kit Enrichment beads. Using the NEBNext Ultra II DNA Library Prep Kit (New England Biolabs) for end repair, a-tailing, and adapter ligation. This uses a custom protocol which resembles the standard NEBNext Ultra II DNA Library Prep protocol but where library preparation occurs while DNA is bound to the Enrichment beads. For library amplification, 10 to 16 PCR cycles were required, determined by the sample biotinylation percentage. The Hi-C sequencing was performed using paired-end sequencing with a read length of 150 bp on an Illumina NovaSeq 6000 instrument.

### Genome assembly, curation and evaluation

***Assembly***

Prior to assembly of the PacBio HiFi reads, a database of *k*-mer counts ( *k* = 31) was generated from the filtered reads using FastK. GenomeScope2 ( Ranallo-Benavidez *et al.*, 2020) was used to analyse the *k*-mer frequency distributions, providing estimates of genome size, heterozygosity, and repeat content.

The HiFi reads were first assembled using Hicanu ( Nurk *et al.*, 2020). Haplotypic duplications were identified and removed using purge_dups ( Guan *et al.*, 2020). The Hi-C reads were mapped to the primary contigs using bwa-mem2 ( Vasimuddin *et al.*, 2019). The contigs were further scaffolded using the provided Hi-C data ( Rao *et al.*, 2014) in YaHS ( Zhou *et al.*, 2023) using the --break option for handling potential misassemblies. The scaffolded assemblies were evaluated using Gfastats ( Formenti *et al.*, 2022), BUSCO ( Manni *et al.*, 2021) and MERQURY.FK ( Rhie *et al.*, 2020).

The mitochondrial genome was assembled using MitoHiFi ( Uliano-Silva *et al.*, 2023), which runs MitoFinder ( Allio *et al.*, 2020) and uses these annotations to select the final mitochondrial contig and to ensure the general quality of the sequence.

***Assembly curation***

The assembly was decontaminated using the Assembly Screen for Cobionts and Contaminants (ASCC) pipeline (article in preparation). Flat files and maps used in curation were generated in TreeVal ( Pointon *et al.*, 2023). Manual curation was primarily conducted using PretextView ( Harry, 2022), with additional insights provided by JBrowse2 ( Diesh *et al.*, 2023) and HiGlass ( Kerpedjiev *et al.*, 2018). Scaffolds were visually inspected and corrected as described by Howe *et al*. (2021). Any identified contamination, missed joins, and mis-joins were corrected, and duplicate sequences were tagged and removed. Sex chromosomes were identified based on read coverage statistics. The curation process is documented at https://gitlab.com/wtsi-grit/rapid-curation (article in preparation).

***Assembly quality assessment***

The Merqury.FK tool ( Rhie *et al.*, 2020), run in a Singularity container ( Kurtzer *et al.*, 2017), was used to evaluate *k*-mer completeness and assembly quality for the primary and alternate haplotypes using the *k*-mer databases ( *k* = 31) that were computed prior to genome assembly. The analysis outputs included assembly QV scores and completeness statistics.

A Hi-C contact map was produced for the final version of the assembly. The Hi-C reads were aligned using bwa-mem2 ( Vasimuddin *et al.*, 2019) and the alignment files were combined using SAMtools ( Danecek *et al.*, 2021). The Hi-C alignments were converted into a contact map using BEDTools ( Quinlan & Hall, 2010) and the Cooler tool suite ( Abdennur & Mirny, 2020). The contact map is visualised in HiGlass ( Kerpedjiev *et al.*, 2018).

The blobtoolkit pipeline is a Nextflow port of the previous Snakemake Blobtoolkit pipeline ( Challis *et al.*, 2020). It aligns the PacBio reads in SAMtools and minimap2 ( Li, 2018) and generates coverage tracks for regions of fixed size. In parallel, it queries the GoaT database ( Challis *et al.*, 2023) to identify all matching BUSCO lineages to run BUSCO ( Manni *et al.*, 2021). For the three domain-level BUSCO lineages, the pipeline aligns the BUSCO genes to the UniProt Reference Proteomes database ( Bateman *et al.*, 2023) with DIAMOND blastp ( Buchfink *et al.*, 2021). The genome is also divided into chunks according to the density of the BUSCO genes from the closest taxonomic lineage, and each chunk is aligned to the UniProt Reference Proteomes database using DIAMOND blastx. Genome sequences without a hit are chunked using seqtk and aligned to the NT database with blastn ( Altschul *et al.*, 1990). The blobtools suite combines all these outputs into a blobdir for visualisation.

The blobtoolkit pipeline was developed using nf-core tooling ( Ewels *et al.*, 2020) and MultiQC ( Ewels *et al.*, 2016), relying on the Conda package manager, the Bioconda initiative ( Grüning *et al.*, 2018), the Biocontainers infrastructure ( da Veiga Leprevost *et al.*, 2017), as well as the Docker ( Merkel, 2014) and Singularity ( Kurtzer *et al.*, 2017) containerisation solutions.

Table 4 contains a list of relevant software tool versions and sources.

**Table 4.** Software tools: versions and sources.

Software tool	Version	Source
BEDTools	2.30.0	https://github.com/arq5x/bedtools2
BLAST	2.14.0	ftp://ftp.ncbi.nlm.nih.gov/blast/executables/blast+/
BlobToolKit	4.3.9	https://github.com/blobtoolkit/blobtoolkit
BUSCO	5.5.0	https://gitlab.com/ezlab/busco
bwa-mem2	2.2.1	https://github.com/bwa-mem2/bwa-mem2
Cooler	0.8.11	https://github.com/open2c/cooler
DIAMOND	2.1.8	https://github.com/bbuchfink/diamond
fasta_windows	0.2.4	https://github.com/tolkit/fasta_windows
FastK	427104ea91c78c3b8b8b49f1a7d6bbeaa869ba1c	https://github.com/thegenemyers/FASTK
Gfastats	1.3.6	https://github.com/vgl-hub/gfastats
GoaT CLI	0.2.5	https://github.com/genomehubs/goat-cli
Hicanu	2.2	https://github.com/marbl/canu
HiGlass	44086069ee7d4d3f6f3f0012569789ec138f42b84 aa44357826c0b6753eb28de	https://github.com/higlass/higlass
MerquryFK	d00d98157618f4e8d1a9190026b19b471055b22e	https://github.com/thegenemyers/MERQURY.FK
Minimap2	2.24-r1122	https://github.com/lh3/minimap2
MitoHiFi	2	https://github.com/marcelauliano/MitoHiFi
MultiQC	1.14, 1.17, and 1.18	https://github.com/MultiQC/MultiQC
NCBI Datasets	15.12.0	https://github.com/ncbi/datasets
Nextflow	23.04.1	https://github.com/nextflow-io/nextflow
PretextView	0.2	https://github.com/sanger-tol/PretextView
purge_dups	1.2.3	https://github.com/dfguan/purge_dups
samtools	1.19.2	https://github.com/samtools/samtools
sanger-tol/ascc	-	https://github.com/sanger-tol/ascc
sanger-tol/blobtoolkit	0.5.1	https://github.com/sanger-tol/blobtoolkit
Seqtk	1.3	https://github.com/lh3/seqtk
Singularity	3.9.0	https://github.com/sylabs/singularity
TreeVal	1.2.0	https://github.com/sanger-tol/treeval
YaHS	yahs-1.1.91eebc2	https://github.com/c-zhou/yahs

### Genome annotation

The Ensembl Genebuild annotation system ( Aken *et al.*, 2016) was used to generate annotation for the *Cryptocephalus primarius* assembly (GCA_963576515.1) in Ensembl Rapid Release at the EBI. Annotation was created primarily through alignment of transcriptomic data to the genome, with gap filling via protein-to-genome alignments of a select set of proteins from UniProt ( UniProt Consortium, 2019).

### Wellcome Sanger Institute – Legal and Governance

The materials that have contributed to this genome note have been supplied by a Darwin Tree of Life Partner. The submission of materials by a Darwin Tree of Life Partner is subject to the **‘Darwin Tree of Life Project Sampling Code of Practice’**, which can be found in full on the Darwin Tree of Life website here. By agreeing with and signing up to the Sampling Code of Practice, the Darwin Tree of Life Partner agrees they will meet the legal and ethical requirements and standards set out within this document in respect of all samples acquired for, and supplied to, the Darwin Tree of Life Project.

Further, the Wellcome Sanger Institute employs a process whereby due diligence is carried out proportionate to the nature of the materials themselves, and the circumstances under which they have been/are to be collected and provided for use. The purpose of this is to address and mitigate any potential legal and/or ethical implications of receipt and use of the materials as part of the research project, and to ensure that in doing so we align with best practice wherever possible. The overarching areas of consideration are:

•   Ethical review of provenance and sourcing of the material

•   Legality of collection, transfer and use (national and international)

Each transfer of samples is further undertaken according to a Research Collaboration Agreement or Material Transfer Agreement entered into by the Darwin Tree of Life Partner, Genome Research Limited (operating as the Wellcome Sanger Institute), and in some circumstances other Darwin Tree of Life collaborators.

## Data Availability

European Nucleotide Archive: Cryptocephalus primarius. Accession number PRJEB56483; https://identifiers.org/ena.embl/PRJEB56483. The genome sequence is released openly for reuse. The *Cryptocephalus primarius* genome sequencing initiative is part of the Darwin Tree of Life (DToL) project. All raw sequence data and the assembly have been deposited in INSDC databases. Raw data and assembly accession identifiers are reported in Table 1 and Table 2.

## Author information

Members of the Natural History Museum Genome Acquisition Lab are listed here: https://doi.org/10.5281/zenodo.12159242.

Members of the Darwin Tree of Life Barcoding collective are listed here: https://doi.org/10.5281/zenodo.12158331.

Members of the Wellcome Sanger Institute Tree of Life Management, Samples and Laboratory team are listed here: https://doi.org/10.5281/zenodo.12162482.

Members of Wellcome Sanger Institute Scientific Operations: Sequencing Operations are listed here: https://doi.org/10.5281/zenodo.12165051.

Members of the Wellcome Sanger Institute Tree of Life Core Informatics team are listed here: https://doi.org/10.5281/zenodo.12160324.

Members of the Tree of Life Core Informatics collective are listed here: https://doi.org/10.5281/zenodo.12205391.

Members of the Darwin Tree of Life Consortium are listed here: https://doi.org/10.5281/zenodo.4783558.

## References

[ref-1] Abdennur N Mirny LA : Cooler: scalable storage for Hi-C data and other genomically labeled arrays. *Bioinformatics.* 2020;36(1):311–316. 10.1093/bioinformatics/btz540 31290943 PMC8205516

[ref-2] Aken BL Ayling S Barrell D : The Ensembl gene annotation system. *Database (Oxford).* 2016;2016: baw093. 10.1093/database/baw093 27337980 PMC4919035

[ref-3] Allio R Schomaker-Bastos A Romiguier J : MitoFinder: efficient automated large-scale extraction of mitogenomic data in target enrichment phylogenomics. *Mol Ecol Resour.* 2020;20(4):892–905. 10.1111/1755-0998.13160 32243090 PMC7497042

[ref-4] Altschul SF Gish W Miller W : Basic local alignment search tool. *J Mol Biol.* 1990;215(3):403–410. 10.1016/S0022-2836(05)80360-2 2231712

[ref-5] Back from the Brink: Species summary. *Rock-rose Pot Beetle*. 2022. Reference Source

[ref-6] Bateman A Martin MJ Orchard S : UniProt: the universal protein knowledgebase in 2023. *Nucleic Acids Res.* 2023;51(D1):D523–D531. 10.1093/nar/gkac1052 36408920 PMC9825514

[ref-7] Bates A Clayton-Lucey I Howard C : Sanger Tree of Life HMW DNA fragmentation: diagenode Megaruptor ^®^3 for LI PacBio. *protocols.io.* 2023. 10.17504/protocols.io.81wgbxzq3lpk/v1

[ref-8] Beasley J Uhl R Forrest LL : DNA barcoding SOPs for the Darwin Tree of Life project. *protocols.io.* 2023; [Accessed 25 June 2024]. 10.17504/protocols.io.261ged91jv47/v1

[ref-9] Bezděk J Sekerka L : Catalogue of palaearctic coleoptera. Volume 6/2/1. Updated and revised second edition. Chrysomeloidea II (Orsodacnidae, Megalopodidae, Chrysomelidae).Leiden: Brill,2024. 10.1163/9789004443303

[ref-10] Buchfink B Reuter K Drost HG : Sensitive protein alignments at Tree-of-Life scale using DIAMOND. *Nat Methods.* 2021;18(4):366–368. 10.1038/s41592-021-01101-x 33828273 PMC8026399

[ref-11] Challis R Kumar S Sotero-Caio C : Genomes on a Tree (GoaT): a versatile, scalable search engine for genomic and sequencing project metadata across the eukaryotic Tree of Life [version 1; peer review: 2 approved]. *Wellcome Open Res.* 2023;8:24. 10.12688/wellcomeopenres.18658.1 36864925 PMC9971660

[ref-12] Challis R Richards E Rajan J : BlobToolKit – interactive quality assessment of genome assemblies. *G3 (Bethesda).* 2020;10(4):1361–1374. 10.1534/g3.119.400908 32071071 PMC7144090

[ref-13] Cox ML : Atlas of the seed and leaf beetles of Britain.Newbury: Pisces Publications,2007. Reference Source

[ref-14] Crowley L Allen H Barnes I : A sampling strategy for genome sequencing the British terrestrial arthropod fauna [version 1; peer review: 2 approved]. *Wellcome Open Res.* 2023;8:123. 10.12688/wellcomeopenres.18925.1 37408610 PMC10318377

[ref-15] da Veiga Leprevost F Grüning BA Alves Aflitos S : BioContainers: an open-source and community-driven framework for software standardization. *Bioinformatics.* 2017;33(16):2580–2582. 10.1093/bioinformatics/btx192 28379341 PMC5870671

[ref-68] Danecek P Bonfield JK Liddle J : Twelve years of SAMtools and BCFtools. *GigaScience.* 2021;10(2): giab008. 10.1093/gigascience/giab008 33590861 PMC7931819

[ref-16] Denton A Oatley G Cornwell C : Sanger Tree of Life sample homogenisation: PowerMash. *protocols.io.* 2023a. 10.17504/protocols.io.5qpvo3r19v4o/v1

[ref-67] Denton A Yatsenko H Jay J : Sanger Tree of Life wet laboratory protocol collection V.1. *protocols.io.* 2023b. 10.17504/protocols.io.8epv5xxy6g1b/v1

[ref-66] Diesh C Stevens GJ Xie P : JBrowse 2: a modular genome browser with views of synteny and structural variation. *Genome Biol.* 2023;24(1): 74. 10.1186/s13059-023-02914-z 37069644 PMC10108523

[ref-65] Duff AG : Beetles of Britain and Ireland, Volume 4: Cerambycidae to Curculionidae.West Runton: A.G. Duff Publishing,2016. Reference Source

[ref-63] Duff AG : Checklist of beetles of the British Isles.3rd Edition. Iver: Pemberley Books,2018. Reference Source

[ref-21] Ewels P Magnusson M Lundin S : MultiQC: summarize analysis results for multiple tools and samples in a single report. *Bioinformatics.* 2016;32(19):3047–3048. 10.1093/bioinformatics/btw354 27312411 PMC5039924

[ref-20] Ewels PA Peltzer A Fillinger S : The nf-core framework for community-curated bioinformatics pipelines. *Nat Biotechnol.* 2020;38(3):276–278. 10.1038/s41587-020-0439-x 32055031

[ref-74] Fagot J : Entretiens sur les Chrysomelidae de Belgique et des régions limitrophes 11 : Les Cryptocephalinae (partim Cryptocephalini) de la faune belge (Coleoptera Chrysomelidae), catalogue et atlas. *Entomologie Faunistique - Faunistic Entomology.* 2020;73:215–239. Reference Source

[ref-160] Formenti G Abueg L Brajuka A : Gfastats: conversion, evaluation and manipulation of genome sequences using assembly graphs. *Bioinformatics.* 2022;38(17):4214–4216. 10.1093/bioinformatics/btac460 35799367 PMC9438950

[ref-24] Grüning B Dale R Sjödin A : Bioconda: sustainable and comprehensive software distribution for the life sciences. *Nat Methods.* 2018;15(7):475–476. 10.1038/s41592-018-0046-7 29967506 PMC11070151

[ref-62] Guan D McCarthy SA Wood J : Identifying and removing haplotypic duplication in primary genome assemblies. *Bioinformatics.* 2020;36(9):2896–2898. 10.1093/bioinformatics/btaa025 31971576 PMC7203741

[ref-61] Harry E : PretextView (Paired REad TEXTure Viewer): a desktop application for viewing pretext contact maps. 2022. Reference Source

[ref-162] Howe K Chow W Collins J : Significantly improving the quality of genome assemblies through curation. *GigaScience.* 2021;10(1): giaa153. 10.1093/gigascience/giaa153 33420778 PMC7794651

[ref-75] Hubble D : A review of the scarce and threatened beetles of Great Britain: the leaf beetles and their allies. 2014. Reference Source

[ref-59] Jay J Yatsenko H Narváez-Gómez JP : Sanger Tree of Life sample preparation: triage and dissection. *protocols.io.* 2023. 10.17504/protocols.io.x54v9prmqg3e/v1

[ref-76] Jolivet P : Biologie des Coléoptères Chrysomélides.Paris: Boubée Publishers,1997. Reference Source

[ref-17] Kerpedjiev P Abdennur N Lekschas F : HiGlass: web-based visual exploration and analysis of genome interaction maps. *Genome Biol.* 2018;19(1): 125. 10.1186/s13059-018-1486-1 30143029 PMC6109259

[ref-18] Kurtzer GM Sochat V Bauer MW : Singularity: scientific containers for mobility of compute. *PLoS One.* 2017;12(5): e0177459. 10.1371/journal.pone.0177459 28494014 PMC5426675

[ref-77] Lawniczak MKN Davey RP Rajan J : Specimen and sample metadata standards for biodiversity genomics: a proposal from the Darwin Tree of Life project [version 1; peer review: 2 approved with reservations]. *Wellcome Open Res.* 2022;7:187. 10.12688/wellcomeopenres.17605.1

[ref-32] Li H : Minimap2: pairwise alignment for nucleotide sequences. *Bioinformatics.* 2018;34(18):3094–3100. 10.1093/bioinformatics/bty191 29750242 PMC6137996

[ref-19] Manni M Berkeley MR Seppey M : BUSCO update: novel and streamlined workflows along with broader and deeper phylogenetic coverage for scoring of eukaryotic, prokaryotic, and viral genomes. *Mol Biol Evol.* 2021;38(10):4647–4654. 10.1093/molbev/msab199 34320186 PMC8476166

[ref-34] Merkel D : Docker: lightweight Linux containers for consistent development and deployment. *Linux J.* 2014;2014(239): 2, [Accessed 2 April 2024]. Reference Source

[ref-38] NBN Atlas Partnership : *Cryptocephalus primarius* Harold, 1872 Rock-rose Pot Beetle map on the NBN Atlas.2024. Reference Source

[ref-78] Nurk S Walenz BP Rhie A : HiCanu: accurate assembly of segmental duplications, satellites, and allelic variants from high-fidelity long reads. *Genome Res.* 2020;30(9):1291–1305. 10.1101/gr.263566.120 32801147 PMC7545148

[ref-39] Oatley G Denton A Howard C : Sanger Tree of Life HMW DNA extraction: automated MagAttract v.2. *protocols.io.* 2023. 10.17504/protocols.io.kxygx3y4dg8j/v1

[ref-79] Owen JA : Observations on the biology of *Cryptocephalus primarius* Harold, 1872 (Coleoptera: Chrysomelidae). *Entomol Gaz.* 2005;56(4):261–269. Reference Source

[ref-80] Pereira L Sivell O Sivess L : DToL taxon-specific standard operating procedure for the terrestrial and freshwater arthropods working group. 2022. 10.17504/protocols.io.261gennyog47/v1

[ref-58] Pointon DL Eagles W Sims Y : sanger-tol/treeval v1.0.0 – Ancient Atlantis. 2023. 10.5281/zenodo.10047654

[ref-22] Quinlan AR Hall IM : BEDTools: a flexible suite of utilities for comparing genomic features. *Bioinformatics.* 2010;26(6):841–842. 10.1093/bioinformatics/btq033 20110278 PMC2832824

[ref-81] Ranallo-Benavidez TR Jaron KS Schatz MC : GenomeScope 2.0 and Smudgeplot for reference-free profiling of polyploid genomes. *Nat Commun.* 2020;11(1): 1432. 10.1038/s41467-020-14998-3 32188846 PMC7080791

[ref-23] Rao SSP Huntley MH Durand NC : A 3D map of the human genome at kilobase resolution reveals principles of chromatin looping. *Cell.* 2014;159(7):1665–1680. 10.1016/j.cell.2014.11.021 25497547 PMC5635824

[ref-82] Rheinheimer J Hassler M : Die Blattkäfer Baden-Württembergs.Karlsruhe: Kleinsteuber Books,2018. Reference Source

[ref-57] Rhie A McCarthy SA Fedrigo O : Towards complete and error-free genome assemblies of all vertebrate species. *Nature.* 2021;592(7856):737–746. 10.1038/s41586-021-03451-0 33911273 PMC8081667

[ref-25] Rhie A Walenz BP Koren S : Merqury: reference-free quality, completeness, and phasing assessment for genome assemblies. *Genome Biol.* 2020;21(1): 245. 10.1186/s13059-020-02134-9 32928274 PMC7488777

[ref-83] Schöller M : Taxonomy of *Cryptocephalus* Geoffroy - what do we know? (Coleoptera: Chrysomelidae: Cryptocephalinae). *Mitteilungen Des Internationalen Entomologischen Vereins.* 2002;28(1/2):59–76. Reference Source

[ref-84] Schöller M : *Cryptocephalus* from Oman and Yemen, with description of a new species and notes on subgenera (Coleoptera: Chrysomelidae). *Mitteilungen Des Internationalen Entomologischen Vereins.* 2021;44(1/2):13–32. Reference Source

[ref-85] Sekerka L Bezděk J Pelikán J : Chrysomelidae s. lat. (mandelinkovití).In: Hejda R., F. J. and Chobot, K. (eds.) *Červený seznam ohrožených druhů České Republiky. Bezobratlí.* 2017;36:308–316.

[ref-86] Sivell O Sivell D Mitchell R : The genome sequence of a leaf beetle, *Cryptocephalus moraei* (Linnaeus, 1758) [version 1; peer review: 2 approved]. *Wellcome Open Res.* 2023;8:467. 10.12688/wellcomeopenres.19522.1 38680651 PMC11053346

[ref-28] Strickland M Cornwell C Howard C : Sanger Tree of Life fragmented DNA clean up: manual SPRI. *protocols.io.* 2023. 10.17504/protocols.io.kxygx3y1dg8j/v1

[ref-87] Telfer MG : *Cryptocephalus ocellatus* Drapiez, 1819 (Chrysomelidae) new to Britain. *Coleopteriste.* 2019a;28(4):155–161. Reference Source

[ref-88] Telfer MG : *Cryptocephalus rufipes* (Goeze, 1777) (Chrysomelidae) new to Britain. *Coleopteriste.* 2019b;28(4):149–154. Reference Source

[ref-29] Twyford AD Beasley J Barnes I : A DNA barcoding framework for taxonomic verification in the Darwin Tree of Life project [version 1; peer review: 2 approved]. *Wellcome Open Res.* 2024;9:339. 10.12688/wellcomeopenres.21143.1 39386966 PMC11462125

[ref-30] Uliano-Silva M Ferreira JGRN Krasheninnikova K : MitoHiFi: a python pipeline for mitochondrial genome assembly from PacBio high fidelity reads. *BMC Bioinformatics.* 2023;24(1): 288. 10.1186/s12859-023-05385-y 37464285 PMC10354987

[ref-31] UniProt Consortium: UniProt: a worldwide hub of protein knowledge. *Nucleic Acids Res.* 2019;47(D1):D506–D515. 10.1093/nar/gky1049 30395287 PMC6323992

[ref-56] Vasimuddin M Misra S Li H : Efficient architecture-aware acceleration of BWA-MEM for multicore systems.In: *2019 IEEE International Parallel and Distributed Processing Symposium (IPDPS).*IEEE,2019;314–324. 10.1109/IPDPS.2019.00041

[ref-89] Warchałowski A : The Palaearctic Chrysomelidae. Identification Keys. Vols. 1 & 2.Warszawa: Natura Optima Dux Foundation,2010. Reference Source

[ref-90] Wiltshire CW Owen JA : The brief history of *Cryptocephalus primarius* Harold (Chrysomelidae) in Britain. *Coleopteriste.* 2004;13(4):162–166. Reference Source

[ref-33] Zhou C McCarthy SA Durbin R : YaHS: Yet another Hi-C Scaffolding tool. *Bioinformatics.* 2023;39(1): btac808. 10.1093/bioinformatics/btac808 36525368 PMC9848053

