# Supplementary material for: The genome sequence of the Rock-rose Pot Beetle, Cryptocephalus primarius Harold, 1872
Source: Wellcome Open Res. 2025 Feb 19;10:77. [Version 1] doi: 10.12688/wellcomeopenres.23703.1 (PMC11986415; doi:10.12688/wellcomeopenres.23703.1)
Supplement: Peer review history

# Reviewer report: Reviewer response for version 1

**DOI:** 10.21956/wellcomeopenres.26149.r120718

**Published:** 2025-04-10

**Authors:** Badr M Al-Shomrani

**Affiliations:** 1 King Abdulaziz City for Science and Technology, Riyadh, Saudi Arabia

The Authors sequenced the genome of an adult male Rock-rose Pot Beetle specimen using PacBio Hi-Fi and Hi-C technologies, generating 2.52 million and 660.92 million reads, respectively. The final assembly demonstrated high quality, with a BUSCO score of 99.1%, a Quality Value of 59.6, and a k-mer completeness of 99.48% for combined primary and alternate assemblies. The methodology was clearly described, and the assembly appears robust.

Are sufficient details of methods and materials provided to allow replication by others?

Yes

Is the rationale for creating the dataset(s) clearly described?

Yes

Are the datasets clearly presented in a useable and accessible format?

Yes

Are the protocols appropriate and is the work technically sound?

Yes

Reviewer Expertise:

Bioinformatics

I confirm that I have read this submission and believe that I have an appropriate level of expertise to confirm that it is of an acceptable scientific standard.

# Reviewer report: Reviewer response for version 1

**DOI:** 10.21956/wellcomeopenres.26149.r120721

**Published:** 2025-04-01

**Authors:** Komal Kumar Raja

**Affiliations:** 1 Baylor College of Medicine, Houston, USA

The manuscript by Mitchell et al. present the genome sequence of the rock-rose pot beetle, *Cryptocephalus primarius*. The authors have extensively introduced the taxonomy, ecology, localization and other aspects of this species. The genome assembly appears impressive with BUSCO of 99.1% and k-mer completeness of 99.48% for the combined. However, the percentage mapped to chromosomes appears a little low. Is this due to repetitive sequences? Can the authors comment on this? The methodology is robust and all bioinformatics tools used in this study have been presented in tables. This allows for reproducibility. Overall, the manuscript and the data is well presented and is a good resource for the scientific community.

Are sufficient details of methods and materials provided to allow replication by others?

Yes

Is the rationale for creating the dataset(s) clearly described?

Yes

Are the datasets clearly presented in a useable and accessible format?

Yes

Are the protocols appropriate and is the work technically sound?

Yes

Reviewer Expertise:

Single cell omics, Bioinformatics, genomics and Neurobiology

I confirm that I have read this submission and believe that I have an appropriate level of expertise to confirm that it is of an acceptable scientific standard.
